# Design of a Hierarchical Control Architecture for Fully-Driven Multi-Fingered Dexterous Hand

**DOI:** 10.3390/biomimetics10070422

**Published:** 2025-06-30

**Authors:** Yinan Jin, Hujiang Wang, Han Ge, Guanjun Bao

**Affiliations:** 1 College of Mechanical Engineering, Zhejiang University of Technology, Hangzhou 310023, China; jynzjut@zjut.edu.cn (Y.J.); 201806041317@zjut.edu.cn (H.W.); g17367117977@163.com (H.G.); 2 ZJUT Yinhu Research Institute of Innovation and Entrepreneurship Fuyang District, Hangzhou 311400, China

**Keywords:** pneumatic artificial muscles, multi-fingered dexterous hand, fuzzy PID

## Abstract

Multi-fingered dexterous hands provide superior dexterity in complex manipulation tasks due to their high degrees of freedom (DOFs) and biomimetic structures. Inspired by the anatomical structure of human tendons and muscles, numerous robotic hands powered by pneumatic artificial muscles (PAMs) have been created to replicate the compliant and adaptable features of biological hands. Nonetheless, PAMs have inherent nonlinear and hysteresis behaviors that create considerable challenges to achieving real-time control accuracy and stability in dexterous hands. In order to address these challenges, this paper proposes a hierarchical control architecture that employs a fuzzy PID strategy to optimize the nonlinear control of pneumatic artificial muscles (PAMs). The FPGA-based hardware integrates a multi-channel digital-to-analog converter (DAC) and a multiplexed acquisition module, facilitating the independent actuation of 20 PAMs and the real-time monitoring of 20 joints. The software implements a fuzzy PID algorithm that dynamically adjusts PID parameters based on both the error and the error rate, thereby effectively managing the nonlinear behaviors of the hand. Experimental results demonstrate that the designed control system achieves high precision in controlling the angle of a single finger joint, with errors maintained within ±1°. In scenarios involving multi-finger cooperative grasping and biomimetic motion demonstrations, the system exhibits excellent synchronization and real-time performance. These results validate the efficacy of the fuzzy PID control strategy and confirm that the proposed system fulfills the precision and stability requirements for complex operational tasks, providing robust support for the application of PAM-driven multi-fingered dexterous hands.

## 1. Introduction

Traditional end-effectors, such as grippers, usually employ low DOF (degree of freedom) mechanisms and rigid actuators, which culminates in their limited functionality and inadequate environmental adaptability. Multi-fingered dexterous hands can realize agile motions with high DOFs through humanoid design with flexible actuators, such as tendon-driven or pneumatic artificial muscle (PAM)-based systems, thereby facilitating compliant manipulation and enhancing environmental adaptability.

As the end-effectors of robots, the performance of multi-fingered dexterous hands depends not only on mechanical design but also on the controlling system. Much attention from researchers have been paid to the hierarchical design paradigm, which is categorized into external and internal configurations according to the deployment of actuators.

Dexterous hands with external actuators (e.g., actuators deployed within the robotic forearm), usually work with tendon transmission. Typically, the Stanford/JPL hand employs a tendon transmission in conjunction with position and force feedback to achieve force-controlled grasping and object shape sensing [1,2]. The Utah/MIT hand utilizes pneumatic actuation and cable transmission, accomplishing grasp planning, motion coordination, and actuator control via a hierarchical control architecture [3,4]. The Robonaut hand incorporates a PowerPC + FPGA hardware architecture, in which the slave controller FPGA processes sensor data and controls actuators [5]. The Shadow Hand integrates a Linux PC for human–machine interface and motion planning, while an embedded PIC microcontroller manages real-time sensor data processing and control, with a CAN bus facilitating bi-directional communication [6,7].

Dexterous hands with embedded actuators, sensors, and controllers in their mechanical framework place a high requirement on structural miniaturization and hardware integration. Representative is the DLR I/II hand, which features a modular mechatronic finger with uniform electromechanical structures. Its control system utilizes a hierarchical architecture, including a global motion coordination layer and a finger-specific control layer [8,9]. The DLR-HIT four-fingered hand employs embedded FPGAs for data acquisition and processing. A DSP + FPGA-based main controller performs real-time control computations, and high-speed serial communication facilitates data exchange and control [10,11]. The Gifu III hand, which possesses 859 tactile detection points, enables comprehensive tactile perception and real-time control on a PC system [12,13]. The Dexhand utilizes a DSP + FPGA hierarchical control design. The FPGA processes sensor data (torque, current, and position), while the DSP executes real-time position and impedance control. Real-time communication between the FPGA and DSP is established via dual-port RAM [14].

The control systems for both categories of dexterous hands typically adopt a three-tier hierarchical architecture of actuation–coordination–planning. In this framework, the intermediate layer is responsible for processing the fundamental actuation and sensor signals through specialized control modules, while concurrently interfacing with the upper computing system to facilitate high-level motion planning.

For externally actuated dexterous hands, flexible actuation is typically employed to mitigate instability resulting from rigid contact with targets. In recent years, various dexterous hands have been developed using soft actuators to enable compliant grasping ability, such as the multi-chamber pneumatic structures employed in the RBO Hand 3 [15]. In a similar vein, the soft smart hand proposed by Yang et al. incorporates granular jamming for stiffness regulation along with embedded position feedback, thereby achieving a harmonious balance between compliance and grasping force output [16]. However, fully soft-actuated dexterous hands encounter challenges of high modelling complexity and low control precision, due to the intrinsic nonlinearity and hysteresis pneumatic artificial muscles (PAMs). Researchers have tried to address this issue by proposing enhanced strategies, including fuzzy PID control [17], feedforward model-based control, adaptive control [18,19], sliding mode control [20,21,22], and neural network-based control [23,24,25].

The integration of hierarchical control with enhanced control strategies constitutes a fundamental approach for augmenting the performance of dexterous hands. Firstly, regarding perception, the multi-fingered dexterous hand necessitates the integration of multimodal sensing systems [26,27], including joint angle, fingertip contact force [28,29,30], visual–tactile sensation [26], and tendon rope tension [31], thereby achieving synchronous acquisition and real-time processing of multi-channel signals. Secondly, concerning actuation, the fully driven multi-fingered dexterous hand, possessing up to 20 degrees of freedom (DOFs), faces significant technical challenges relating to the real-time independent and coordinated control of each driving unit within the control system. Thirdly, with respect to control mechanisms, the input air pressure and output displacement of the pneumatic artificial muscle (PAM) exhibit nonlinear characteristics; therefore, it is imperative to design a robust control algorithm that establishes equilibrium between dynamic response characteristics and system stability.

In recent years, bio-inspired design has emerged as a key driver for the development of novel robotic systems [32]. Researchers continue to draw inspiration from the structure and function of natural organisms to develop biomimetic mechanical systems characterized by both structural flexibility and functional dexterity, with applications spanning from biomimetic wings [33] to soft robots. Inspired by soft organisms in nature, a research group at Technische Universität München [34] has developed a silicon elastomeric tentacle actuated by an embedded pneumatic channel, capable of securely gripping and transporting objects weighing up to 5 kg. The proposed pneumatic tentacle features a straightforward control mechanism and demonstrates considerable versatility [35]. Consequently, it holds promising potential applications within the field of robotics. Saint Petersburg Electrotechnical University [35] also published details of a tentacle robot which has good energy efficiency. Load cells and stepper motors are utilized in their novel design concept to solve the problems of positioning and motion control. A collaborative research team [36] from the University of Science and Technology Beijing and Queen Mary University of London has proposed a cable-driven soft robot inspired by the octopus tentacle. The dynamic equation derived from the extended Hamilton’s principle, the boundary control strategy developed through Lyapunov-based design, and the stability analysis utilizing LaSalle’s invariance principle are employed to demonstrate the applicability and effectiveness of the proposed distributed parameter modelling and control strategy, which is capable of being promoted within the field of soft robotics [36]. Compared to the bio-inspired robots previously examined, hand-type robots typically possess a greater number of degrees of freedom and are more proficient in replicating the structure and movements of the human hand. Consequently, they achieve superior manipulation capabilities. Furthermore, the primary objective of their design is to emulate the flexibility and sensory perception capabilities of human hands to the greatest extent feasible.

In the study of multi-fingered dexterous hands, Pneumatic Artificial Muscle (PAM) has become a prominent choice for realizing flexible actuation and bionic movement control, owing to its contraction movement pattern that closely resembles biological muscles, along with its advantageous combination of flexibility and a high force-to-weight ratio. At the levels of patent and industrial development, open patents such as DE102019208773A1 and WO2023217935A1 indicate that both academia and industry have maintained a sustained interest in the research of air-driven flexible actuators. On the commercial front, although notable platforms such as Shadow Hand and DLR-HIT Hand have accomplished the realization of complex hand structures and multi-degree-of-freedom control, the majority still utilize traditional motor-driven architectures, which hinder the balance between bionics and flexibility in terms of control accuracy and responsiveness. Under the current guidance of the bionic design concept, the design of a bionic multi-finger dexterous hand system, founded on pneumatic artificial muscles, alongside the supportive development of a real-time, non-linear adaptive, and highly integrated control architecture, not only addresses the technical challenges faced by the academic community concerning the high-efficiency control of flexible actuators but also presents a novel solution for the industry regarding the practical implementation of soft bionic hand engineering concepts. The contributions of this paper are primarily evident in three aspects: the design of control system architecture, the optimization of nonlinear control strategies for pneumatic artificial muscles (PAMs), and the development of human–machine interaction interfaces.

1. Informed by the modular design principles applicable to both hardware and software, a layered control system architecture is presented to develop a four-channel high-precision digital-to-analog converter (DAC) driver module, utilizing a field-programmable gate array (FPGA) as the real-time master control unit. This system is integrated with a multiplexed signal acquisition module to facilitate the control and sensing capabilities of a fully-driven multi-fingered dexterous hand, while simultaneously ensuring the real-time performance of the system.
2. In addressing the hysteresis nonlinear characteristics of the PAM, we employ a fuzzy PID control strategy. This approach utilizes fuzzy control rules alongside an offline table checking method to swiftly adjust the PID parameters. Consequently, it enhances the time-varying adaptive capacity of the PAM driving system, thereby improving both the real-time performance and robustness of the system. In contrast to traditional fuzzy PID control, predominantly utilized in single-degree-of-freedom systems or simulation platforms, this study, for the first time, applies a real-time control application aimed at a 20-channel high-degree-of-freedom PAM-driven system. This implementation showcases the practicality and scalability of the method in intricate nonlinear contexts.
3. Develop the upper computer control terminal utilizing the Qt framework, integrate the three-dimensional model reconstruction module, and implement multiple operational modes and state monitoring through the visualization interface, facilitated by forward and inverse kinematic algorithms.

## 2. System Architecture

### 2.1. Multi-Fingered Dexterous Hand Design

The mechanical structure of the dexterous hand utilized in this study is based on our previous research presented at the 2022 International Conference on Advanced Robotics and Mechatronics [37]. During this conference, a modular full-drive multi-fingered dexterous hand was developed. This research highlighted the tendon-driven mechanical design, which incorporates unilateral tendon actuation, integrated torsion and tension spring return mechanisms, along with joint modules inspired by the anatomical and kinematic characteristics of the human hand.

**Figure 1:**
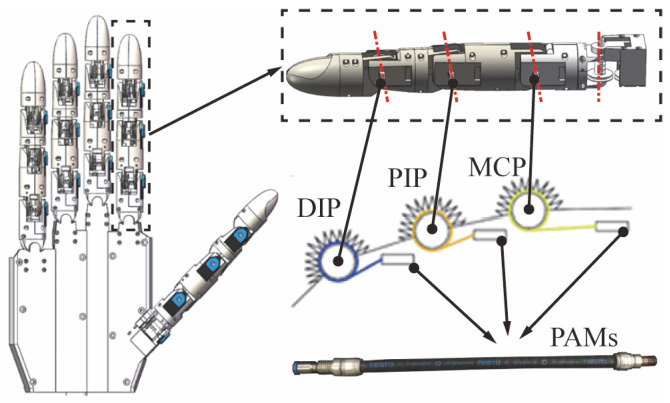
Dexterous hand structure.

The dexterous hand is comprised of five fingers, each consisting of three bending joints and one lateral swing joint, yielding a cumulative total of 20 degrees of freedom (DOFs), as depicted in Figure 1. The joint types include the Distal Interphalangeal Joint (DIP), Proximal Interphalangeal Joint (PIP), and Metacarpophalangeal Joint (MCP), organized to emulate human finger articulation.

The skilled hand utilizes pneumatic artificial muscles, which resemble the structure of the human physiological drive system, serving as the actuator. Pneumatic artificial muscles not only endow the fingers with flexible properties but also improve their adaptability to complex operating environments [38,39]. Due to the substantial dimensions of the pneumatic artificial muscles, a combination of external driving forces and tendon transmission is employed. In comparison to conventional transmission methods, tendon transmission facilitates joint movement through unilateral tension, along with the synergistic return action of tension and torsion springs, thereby significantly simplifying the mechanical structure.

To achieve complete functionality, it is essential to configure 20 pneumatic muscles along with their corresponding tendon ropes. Such a configuration necessitates stringent requirements regarding the real-time and multi-unit synergy of both the control system software and hardware.

In contrast to our previous work, which concentrated on the mechanical design and fundamental kinematic structure, this paper emphasizes the design and implementation of the control system, particularly focusing on the development of a real-time fuzzy PID control strategy, integration of hardware and software on FPGA, and system-level validation through multi-mode experimentation.

### 2.2. Hardware Modular Design

In order to meet the functional requirements of the fully-driven multi-fingered dexterous hand, which encompasses the independent actuation of pneumatic artificial muscles, comprehensive degrees of freedom sensing, and real-time data transmission, the hardware of this control system must fulfill the following three essential functionalities:

1. Independent actuation of twenty pneumatic artificial muscles.
2. The acquisition of twenty joint angle signals along with five fingertip force sensor signals.
3. The real-time transmission of sensor data to the host computer facilitates the visualization of the dexterous hand’s motion state.

**Figure 2:**
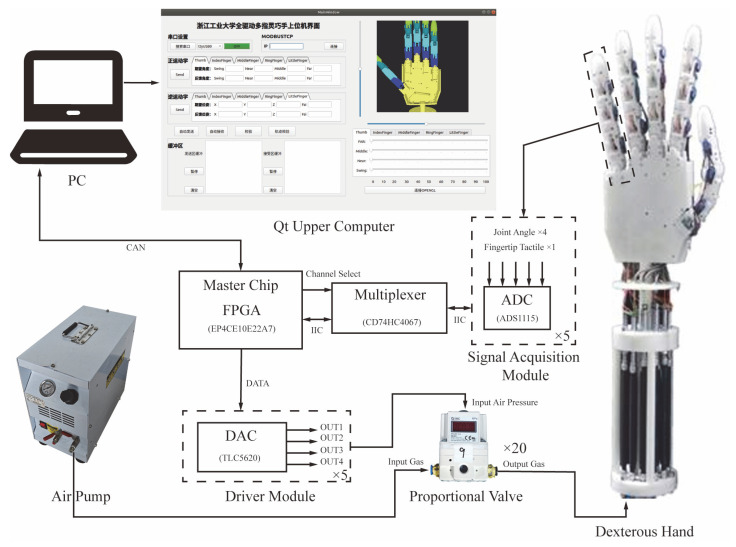
Hardware design. The Chinese content utilized in the Qt Upper Computer section of the figure illustrates a proprietary upper computer software interface. This interface allows for the input of forward or inverse kinematics parameters to operate the developed dexterous hand.

The system hardware utilizes a modular design, as illustrated in Figure 2, to improve the system’s expandability, reliability, and maintainability. The primary control module is founded on an FPGA chip (model: EP4CE10E22A7), capitalizing on its parallel processing capabilities to execute drive control, signal acquisition, and data transmission tasks in real-time.

The primary control chip is capable of transmitting only digital signals. In contrast, the final regulation of the pneumatic muscles of the electrical proportional valve necessitates analog voltage for control purposes. Consequently, the primary control chip initially dispatches digital signals to the driver module, which employs a DAC chip (model: TLC5620) to convert these digital signals into analog voltage signals for input to the electric proportional valve. Electrical proportional valves modulate the air pressure based on the input voltage, thereby actuating the pneumatic artificial muscles (Festo DMSP-5-AM-CM) to facilitate joint movements. Given that the system is required to drive 20 pneumatic artificial muscles, a total of 20 electrical proportional valves are configured. It is noteworthy that the TLC5620 DAC chip provides only four output channels; therefore, the driver module utilizes 5 DAC chips in a cascading arrangement to achieve 20 control signal outputs.

The sensor module includes 20 joint angle sensors (model: SV01A103, 4 per finger) and 5 fingertip force sensors (1 per finger). The angle sensors employed are SV01A103 contact-based rotary displacement sensors, which offer stable and responsive measurements that are suitable for real-time joint control. In order to minimize the coupling of the hardware system, the module employs a multiplexer to timely acquire the sensor signals. The four angular signals and one tactile signal from each finger are obtained by a single ADC chip and subsequently transmitted to the main control chip via the IIC bus.

To achieve the real-time visualization of the dexterous hand movement status, the master control module transmits sensor data to the host computer via the CAN bus. Moreover, the system is equipped with an independent power supply module, which provides a stable power supply for the FPGA, electrical proportional valve, DAC, and other modules through a multi-stage voltage regulator and voltage reduction circuit.

### 2.3. Hierarchical Control Design

**Figure 3:**
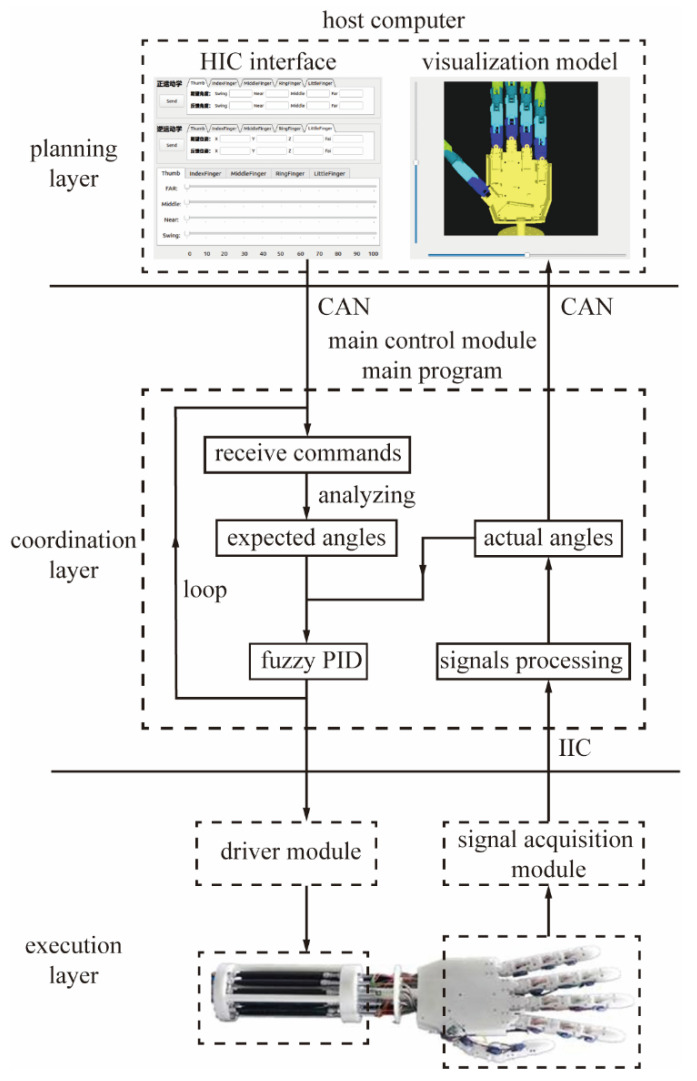
Three-layer hierarchical structure. The Chinese content utilized in the HIC interface section of the figure illustrates a proprietary upper computer software interface. This interface allows for the input of forward or inverse kinematics parameters to operate the developed dexterous hand.

In order to enhance the development efficiency, readability, and maintainability of the control system, and in accordance with the design principle of ‘high cohesion and low coupling’, the control system employs a three-layer hierarchical architecture, as Figure 3 shows:

- Top layer—Planning Layer (Host Computer):

The host computer is tasked with the responsibility of recording and visualizing the sensor data, as well as providing a real-time display of the movement status of the dexterous hand. Furthermore, the host computer receives user input via the human–computer interaction interface, integrates this input with the trajectory planning algorithm to generate the target trajectory, and subsequently transmits the commands to the lower computer.

- Intermediate Layer—Coordination Layer (Main Control Module):

The intermediate layer is responsible for receiving commands from the host computer and allocating each driver’s control signals according to the specified target angle value utilizing a fuzzy Proportional-Integral-Derivative (PID) control algorithm. Furthermore, the intermediate layer is tasked with processing the underlying sensor data and transmitting the processed information to the host computer.

- Bottom layer—Execution Layer (Driver and Acquisition):

The bottom layer comprises the driver module and the signal acquisition module. The driver module is tasked with converting digital control signals into corresponding analog voltage values, subsequently activating the pneumatic artificial muscles via the electrical proportional valves, thereby facilitating the precise control of the joints. Concurrently, the signal acquisition module is responsible for gathering the joint angle and tactile sensor signals, which are then transmitted to the host computer through the main control module.

### 2.4. Main Control Module Design

The software system is constructed with a distributed architecture and is segmented into two primary functional units: the host computer and the main control board. Within this framework, the main control board serves as the execution core of the system, tasked with receiving commands from the host computer, processing sensor data, executing control algorithms, and actuating pneumatic muscles. The principal program flow is delineated as follows:

1. The primary program concludes the hardware initialization process, which encompasses the communication interface, sensor, and driver module, following startup. Thereafter, it transitions into standby mode to continuously monitor commands from the host computer in real-time.
2. Upon receiving the anticipated angle commands from the host computer, an analysis of the target driver module and the output port address is conducted via the communication protocol to ensure the precise assignment of commands.
3. Simultaneously, the actual joint angle signals are acquired and transformed into digital signals by the ADS1115 analog-to-digital converter chip. These digital signals are subsequently processed by the main control module and relayed to the host computer for real-time display.
4. The fuzzy PID controller analyzes the anticipated and actual angle data to compute drive signals for accurate joint control.
5. The digital drive signals are spliced and transmitted to the driver module, which actuates the electric proportional valve to regulate air pressure and facilitate joint movement.
6. The program implements the aforementioned process in a predetermined cycle to ensure the stability of real-time and closed-loop control.

## 3. Control Strategy

**Figure 4:**
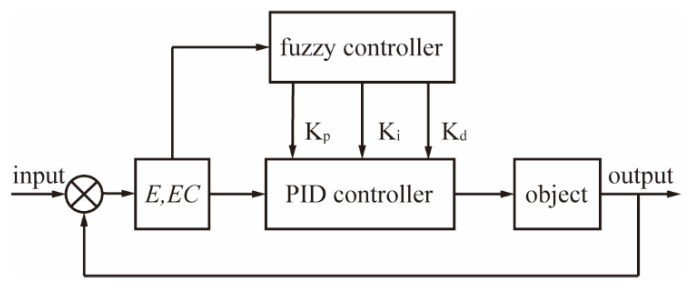
Fuzzy PID control principle.

The PID algorithm is widely used in the control field due to its simple structure, high versatility, and robustness, especially for linear control systems that can be accurately modeled mathematically [40,41]. However, in this control system, the relationship between the voltage control value of the electric proportional valve and the joint angle value in the final dexterous hand is nonlinear, and the characteristics of different joints vary. As Figure 4 shows, to address the challenge that the traditional PID algorithm faces in controlling nonlinear systems, this paper proposes a fuzzy PID control strategy that integrates fuzzy logic with the PID algorithm [42,43]. The PID parameters are dynamically adjusted based on the error (*E*) and the error change rate (*EC*) to achieve effective control of the nonlinear system.

The expression for incremental PID control is articulated as follows:

(1) $$∆u\left(t\right)=K_{p} [e\left(t\right)-e\left(t-1\right)]+K_{i}\cdot e\left(t\right)+K_{d} [e\left(t\right)-2e\left(t-1\right)+e\left(t-2\right)]$$

where $∆u\left(t\right)$ is the control increment, $e\left(t\right)$ is the current error, and $K_{p}$, $K_{i}$ and $K_{d}$ are the proportional, integral and differential gains, respectively.

The fuzzy PID controller adjusts to the nonlinear characteristics of the system by dynamically modifying the $K_{p}$, $K_{i}$ and $K_{d}$ parameters. The fuzzy rules are based on the real-time error (E) and the error change rate (EC) of the joint angles, and a seven-level discrete domain is used, defined as {NB, NM, NS, ZO, PS, PM, PB}, which corresponds to negative large, negative medium, negative small, zero, positive small, positive medium, and positive large, respectively.

The selection of proportional gain has a direct impact on the response speed and overshoot of the system. An increased value of $K_{p}$ can enhance the system’s response speed; however, it may also result in a higher overshoot, consequently affecting system stability. Conversely, a reduced value of $K_{p}$ lessens the overshoot but results in a slower response speed and an extended control time. Considering the influence of $K_{p}$ on the system, the fuzzy control rules are outlined in Table 1. During the initial phase of the system response, a higher $K_{p}$ value is chosen to enhance response speed. In the transition phase, the $K_{p}$ value is judiciously decreased to minimize overshoot. Lastly, in the steady-state phase, the $K_{p}$ value is appropriately increased to mitigate steady-state deviation and enhance control accuracy.

Table 2 demonstrates that the selection of the integral gain denoted as $K_{i}$ significantly influences the effectiveness of the system’s steady-state bias cancellation. An increased value of $K_{i}$ may result in integral saturation and overshoot, whereas a decreased value of $K_{i}$ will extend the control time. Considering the impact of $K_{i}$ on the system, the fuzzy control rules are delineated as follows: during the initial stage, the value of $K_{i}$ should be judiciously reduced to avert the saturation of the integrals; in the transition stage, the value of $K_{i}$ should be appropriately elevated to augment the effect of the integrals; and in the steady state stage, the value of $K_{i}$ is to be further increased to minimize the steady-state deviation and enhance control accuracy.

The selection of the differential gain $K_{d}$ significantly impacts the dynamic characteristics of the system, as Table 3 indicates. An increase in the value of $K_{d}$ can mitigate overshoot; however, it may also prolong the adjustment time. Conversely, a decrease in the value of $K_{d}$ may amplify system oscillations. Considering the effects of $K_{d}$ on the system, the fuzzy control rules are outlined as follows: during the initial phase of the system, a higher value of $K_{d}$ is chosen to minimize overshoot; in the transition phase, the value of $K_{d}$ remains constant or is reduced appropriately; and in the steady phase, the value of $K_{d}$ is further decreased to lessen the braking effect and optimize adjustment time.

The outputs of the target objects derived from the fuzzy inference module require conversion into specific physical quantities through the process of defuzzification. In this paper, the triangular membership function is employed, and defuzzification is conducted using the center of gravity method:

(2) $$V_{0}=\frac{\sum_{i=0}^{n}M_{i}\times F_{i}}{\sum_{i=0}^{n}M_{i}}$$

where $M_{i}$ is the degree of membership and $F_{i}$ is the fuzzy quantization value.

To mitigate real-time computational overhead and ensure deterministic timing within the control process, this paper employs an offline fuzzy PID design method utilizing ROM lookup tables. The following delineates the specific implementation steps:

1. Rule table generation and quantization: The fuzzy control logic, which encompasses membership functions, linguistic levels, fuzzy inference rules, and the center-of-gravity defuzzification method, was meticulously designed utilizing Fuzzy Logic Toolbox of MATLAB 2022. All parameter increments (Δ*Kₚ*, Δ*Kᵢ*, Δ*K_d_*) were represented utilizing 8-bit signed integers (−127 to +127) to facilitate fixed-point arithmetic. Overflow saturation logic was incorporated to avert wraparound, and rounding was executed by truncation to ensure numerical stability and deterministic control timing.
2. Address encoding and ROM formatting: The entries of the fuzzy rule table were encoded utilizing 8-bit addressing: the high 4 bits denote the fuzzy level of *E*, while the low 4 bits indicate that of *EC*. The quantized rule table was exported in hexadecimal format and subsequently embedded into the FPGA ROM through a Memory Initialization File (MIF).
3. Hardware implementation: The control logic was implemented using Verilog HDL and synthesized with the Quartus Prime Lite Edition published by Intel Corporation located at Santa Clara of the USA, specifically targeting the Altera Cyclone IV EP4CE10E22A7 chip developed by the same company. The ROM blocks were initialized with the MIF file during the synthesis process. Within the control cycle, the system performs a direct lookup in the ROM according to the current values of *E* and *EC*, retrieving the corresponding parameter increments.
4. Control logic execution: The retrieved increments $K_{p}$, $K_{i}$ and $K_{d}$ are utilized in the incremental PID computation module to facilitate dynamic updates of the PID gains. Overflow protection and rounding logic have been integrated through saturation and truncation methods, ensuring numerical stability and consistent execution within the FPGA environment.

This ROM-based fuzzy PID control architecture effectively circumvents runtime fuzzy inference computation, substantially mitigates on-chip processing load, and enhances both response speed and control accuracy for the dexterous hand.

## 4. Experiments and Results

In order to verify the effectiveness of the control system developed in this paper, as well as the proposed fuzzy PID control strategy within the pneumatic muscle-driven multi-fingered dexterous hand system, a series of experiments has been meticulously designed. These experiments encompass single-finger joint angle control, collaborative grasping using a multi-fingered dexterous hand, and bionic movement demonstrations. The objective of the experiments is to validate the accuracy, stability, and adaptability of the control system under various operating conditions, while also assessing the control performance of the fuzzy PID algorithm in nonlinear systems.

### 4.1. Joint-Level Control of Finger

**Figure 5:**
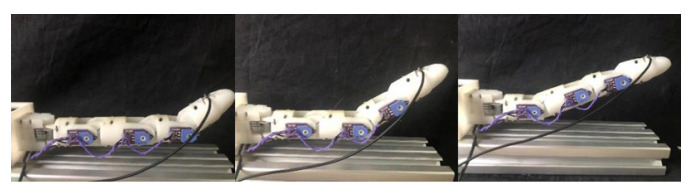
Joint-level control of finger.

The experiment on single-finger joint angle control primarily concentrates on the distal, middle, and proximal joints of the individual finger. In consideration of the range of motion of the finger, this experiment establishes the anticipated angular presets of 30°, 60°, and 80°, as Figure 5 shows. The host computer receives feedback data from angle sensors and conducts a comparison with the actual joint angles.

The experimental results, listed in Table 4, indicate that the bending angle error for each joint is maintained within ±1°, indicating that the designed control system exhibits a high degree of angle control accuracy. The potential source of the error may involve the elastic decay and relaxation of the pneumatic artificial muscles (PAMs) and tendons during repeated usage. Furthermore, the noise interference in the sensor signal and the hysteresis characteristics of the pneumatic muscle could also impact the control accuracy.

### 4.2. Bionic Movement of Multi-Fingered Dexterous Hand

**Figure 6:**
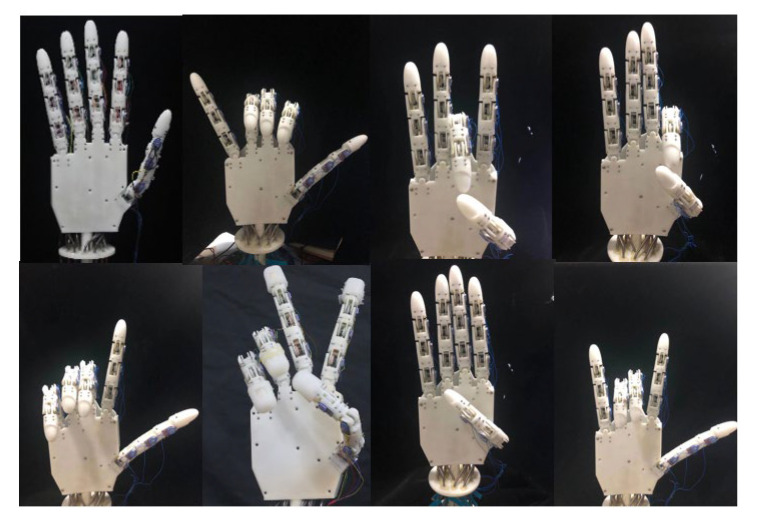
Gesture control experiment.

The experiment involving the bionic movement of a multi-fingered dexterous hand, as Figure 6 exhibits, is conducted to evaluate the synchronization and real-time performance of the control system, as well as the efficacy of coordinated multi-finger movements. By executing grasping operations and predefined gesture movements, the experiment assesses both the efficiency and accuracy of the multi-fingered dexterous hand apparatus and its control system throughout the coordinated movement process. The results indicate that the developed control system not only facilitates the interconnection between each finger joint of the multi-fingered dexterous hand apparatus but also enables real-time monitoring of the joint movement data. The algorithm effectively adjusts and compensates for the positions of the finger joints during the movement process, ensuring that the intended movements are executed rapidly and accurately, thereby demonstrating a high degree of coordination and synchronization.

### 4.3. Grasping Performance of Multi-Fingered Dexterous Hand

**Figure 7:**
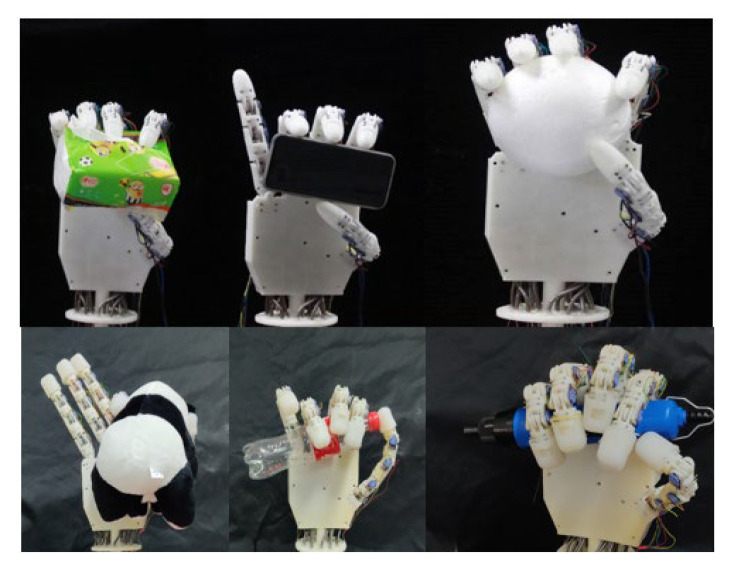
Power grabbing experiment.

To assess the performance of the multi-fingered dexterous hand in practical grasping tasks, a series of grasping experiments were conducted utilizing objects composed of various characteristic materials, as Figure 7 shows. The results of these experiments indicate that the implemented control system is proficient in effectively coordinating the movements of multiple fingers to execute grasping actions for objects of diverse shapes, notwithstanding the considerable size of the fingers. Specifically, the dexterous hand is capable of stabilizing the grasp of rigid, flexible, and irregularly shaped objects, including spheres, rectangles, and cylinders, thereby validating the efficacy of the current control system in managing multi-finger cooperative control.

## 5. Discussion and Conclusions

This paper delineates the design and implementation of a real-time control system for a fully actuated multi-fingered dexterous hand, propelled by pneumatic artificial muscles (PAMs), according to a modular and hierarchical architecture. The efficacy of the proposed system was corroborated through a series of experiments. The primary contributions are summarized as follows:

- A three-layer hierarchical control architecture has been proposed, which features an FPGA-based core controller alongside four-channel DAC modules that facilitate the independent control of 20 PAMs. This system integrates sensor signal acquisition with host communication and demonstrates commendable scalability and maintainability.
- A fuzzy PID control strategy has been developed to manage the nonlinear and hysteretic characteristics of pneumatic artificial muscles (PAMs). By dynamically adjusting the PID parameters in response to error and its rates of change, the controller adeptly addresses complex dynamic conditions and enhances both the stability and flexibility of the system in multi-task scenarios.
- A fully integrated closed-loop system, encompassing sensing, control computation, actuation, and host interaction, has been developed and validated through three distinct experiments: single-joint angle control, multi-finger collaborative grasping, and biomimetic gesture demonstration. The results substantiate the system’s real-time performance, motion accuracy, and synchronization capabilities.

This research establishes a foundation for the implementation of intelligent control strategies in biomimetic dexterous hands. In forthcoming endeavors, our objective is to advance the system towards engineering-oriented iterations, which will entail a comprehensive analysis of cost-performance trade-offs, the formulation of a complete Bill of Materials (BOM), and a quantitative assessment of manufacturability and cost-effectiveness. Furthermore, we will incorporate considerations of sustainability and design feasibility throughout the optimization process.

To enhance precision in control and reduce residual error, we propose the integration of more sophisticated compensation strategies. These strategies include empirical hysteresis modeling, feedforward dynamic prediction modules, and sensor fusion methodologies that integrate joint angle and tendon tension feedback. It is anticipated that these techniques will augment the fuzzy PID controller and improve tracking accuracy within nonlinear actuation dynamics.

In the near future, we plan to benchmark the current fuzzy PID control scheme against alternative control strategies, which include adaptive control, model predictive control (MPC), and methods based on reinforcement learning. This initiative will enable a more thorough evaluation of the performance trade-offs among robustness, real-time feasibility, and control precision across diverse algorithmic paradigms.

Furthermore, to enhance the evaluation of the robustness and generalizability of our system, we aim to formulate and carry out a series of progressively challenging and dynamic task scenarios. These scenarios will incorporate real-time responses to external disturbances, manipulation of objects amidst uncertainty, and operations conducted under suboptimal conditions, which include sensor noise and actuator delay. These experiments will provide a deeper insight into the system’s capability for stable operation in unpredictable environments.

Simultaneously, we will undertake multi-round testing to evaluate the system’s repeatability, reproducibility, and accuracy, which encompasses measurements of standard deviation and maximum deviation. Furthermore, we intend to develop a framework for evaluating durability, concentrating on PAM fatigue performance, component wear, and long-term control reliability during continuous operation.

In continuation of this premise, forthcoming research endeavors will concentrate on the amalgamation of multimodal perception, including visual and tactile sensing, alongside the incorporation of bio-inspired control algorithms, such as imitation learning, to enhance the autonomy and adaptability of the system. This initiative aims to advance towards the subsequent generation of biomimetic robotic hands characterized by human-like manipulation capabilities.

## Figures and Tables

**Table 1:** $K_{p}$ fuzzy rule table.

$\Delta K_{p}$	NB	NM	NS	ZO	PS	PM	PB
NB	PB	PB	PM	PM	PS	ZO	ZO
NM	PB	PB	PM	PS	PS	ZO	NS
NS	PM	PM	PM	PS	ZO	NS	NS
ZO	PM	PM	PS	ZO	NS	NM	NM
PS	PS	PS	ZO	NS	NS	NM	NM
PM	PS	ZO	NS	NM	NM	NM	NB
PB	ZO	ZO	NM	NM	NM	NB	NB

**Table 2:** $K_{i}$ fuzzy rule table.

$\Delta K_{i}$	NB	NM	NS	ZO	PS	PM	PB
NB	NB	NB	NM	NM	NS	ZO	ZO
NM	NB	NB	NM	NS	NS	ZO	ZO
NS	NB	NM	NS	NS	ZO	PS	PS
ZO	NM	NM	NS	ZO	PS	PM	PM
PS	NM	NS	ZO	PS	PS	PM	PB
PM	ZO	ZO	PS	PS	PM	PB	PB
PB	ZO	ZO	PS	PM	PM	PB	PB

**Table 3:** $K_{d}$ fuzzy rule table.

${\Delta K}_{d}$	NB	NM	NS	ZO	PS	PM	PB
NB	PS	NS	NB	NB	NB	NM	PS
NM	PS	NS	NB	NM	NM	NS	ZO
NS	ZO	NS	NM	NM	NS	NS	ZO
ZO	ZO	NS	NS	NS	NS	NS	ZO
PS	ZO	ZO	ZO	ZO	ZO	ZO	ZO
PM	PB	NS	PS	PS	PS	PS	PB
PB	PB	PM	PM	PM	PS	PS	PB

**Table 4:** Single finger joint angle control experiment data sheet.

Joint	Expected Angle (°)	Actual Angle (°)
DIP	30	29.5
	60	60.1
	80	80.4
MCP	30	30.4
	60	60.6
	80	80.1
PIP	30	29.8
	60	59.5
	80	79.7

## Data Availability

The original contributions presented in this study are included in the article. Further inquiries can be directed to the corresponding author.
